# Organic Wheat Farming Improves Grain Zinc Concentration

**DOI:** 10.1371/journal.pone.0160729

**Published:** 2016-08-18

**Authors:** Julian Helfenstein, Isabel Müller, Roman Grüter, Gurbir Bhullar, Lokendra Mandloi, Andreas Papritz, Michael Siegrist, Rainer Schulin, Emmanuel Frossard

**Affiliations:** 1 Institute of Agricultural Sciences, ETH Zurich, 8315, Lindau, Switzerland; 2 Institute of Terrestrial Ecosystems, ETH Zurich, 8092, Zurich, Switzerland; 3 Department of International Cooperation, Research Institute of Organic Agriculture (FiBL), 5070, Frick, Switzerland; 4 Research Division, bioRe Association, Kasrawad, Madhya Pradesh, India; 5 Institute for Environmental Decisions, ETH Zurich, 8092, Zurich, Switzerland; University of Delhi, INDIA

## Abstract

Zinc (Zn) nutrition is of key relevance in India, as a large fraction of the population suffers from Zn malnutrition and many soils contain little plant available Zn. In this study we compared organic and conventional wheat cropping systems with respect to DTPA (diethylene triamine pentaacetic acid)-extractable Zn as a proxy for plant available Zn, yield, and grain Zn concentration. We analyzed soil and wheat grain samples from 30 organic and 30 conventional farms in Madhya Pradesh (central India), and conducted farmer interviews to elucidate sociological and management variables. Total and DTPA-extractable soil Zn concentrations and grain yield (3400 kg ha^-1^) did not differ between the two farming systems, but with 32 and 28 mg kg^-1^ respectively, grain Zn concentrations were higher on organic than conventional farms (*t* = -2.2, *p* = 0.03). Furthermore, multiple linear regression analyses revealed that (a) total soil zinc and sulfur concentrations were the best predictors of DTPA-extractable soil Zn, (b) Olsen phosphate taken as a proxy for available soil phosphorus, exchangeable soil potassium, harvest date, training of farmers in nutrient management, and soil silt content were the best predictors of yield, and (c) yield, Olsen phosphate, grain nitrogen, farmyard manure availability, and the type of cropping system were the best predictors of grain Zn concentration. Results suggested that organic wheat contained more Zn despite same yield level due to higher nutrient efficiency. Higher nutrient efficiency was also seen in organic wheat for P, N and S. The study thus suggests that appropriate farm management can lead to competitive yield and improved Zn concentration in wheat grains on organic farms.

## Introduction

Zinc (Zn) deficiency is a widespread public health problem in India [1–3] and is in large part due to a cereal-dominated diet [3]. It can be seen e.g. in the prevalence of stunted growth in children, which is estimated to affect 61 million children in India under 5 years of age [1]. At the same time, low Zn availability in soils may limit crop yield [4]. In India Zn deficiency is particularly prevalent in Alfisols, Vertisols, Inceptisols, Aridisols, and leached Ultisols [5]. Under low Zn conditions, plant growth is severely reduced and other plant functions are disrupted, as may be expressed in increased susceptibility to diseases [6]. Furthermore, with an annual production of almost 10^11^ kg India is the second biggest producer of wheat globally, with most wheat being produced by the northern and central states of Uttar Pradesh, Punjab, Haryana, and Madhya Pradesh [7]. In these states wheat is also the main staple crop [3]. Research on increasing soil availability and grain Zn concentration in wheat is thus of great importance in India, as it 1) has the potential to alleviate health problems in humans associated with Zn deficiency and 2) may increase agricultural yields.

There are two main approaches for increasing grain Zn concentration: biofortification through crop breeding and agronomic biofortification through Zn fertilizer application [8]. Breeding efforts, led by HarvestPlus and the International Wheat and Maize Improvement Center (CIMMYT), set the goal to increase grain Zn concentration in wheat from 25 to 37 mg kg^-1^ [9]. Several studies have shown that Zn fertilization, especially foliar application, is an effective way to increase grain Zn concentration [2,10]. Ram et al. (2015) reported over 100% increases in wheat grain Zn concentration from 25–33 mg kg^-1^ to 63–70 mg kg^-1^, coupled with significant yield increases, following soil and foliar application of Zn fertilizers on field trials in Punjab [2]. Since grain Zn accumulation is related to soil Zn availability, enhancing soil Zn availability can also increase Zn concentration in cereal grains [8]. A third approach for increasing cereal grain Zn concentrations–that has received much less attention–is biofortification through organic matter management [11].

Together with pH, clay content, and concentrations of other nutrients (especially N and P), organic matter is one of the most important factors determining Zn availability in soils [12,13]. Zn availability may decrease with increasing soil organic matter content, as organic matter binds Zn [14]. However, other studies observed a positive correlation between DTPA (diethylene triamine pentaacetic acid)-extractable Zn and organic matter across different soils, as organic matter may also be a source of exchangeable Zn [5]. Thus it is conceivable that organic matter management may present a possibility for biofortification. While farmyard manure is a major source of Zn in agricultural systems [15], addition of green manure (fresh plant residues) in pot studies was also shown to significantly improve the plant-available fraction of Zn in the soil and increase wheat grain concentrations from 20 to 31 mg kg^-1^ [11].

Do organically grown cereals contain higher concentrations of Zn? Since organic agriculture relies on substantial inputs of organic matter in the form of farmyard manure, compost, or leguminous cover crops, it could potentially increase Zn concentration in the grains. While several studies have looked at yield [16] or economic performance [17] of organic farms, few studies have compared organic and conventional farming in terms of Zn biofortification. Organic farming has been shown to incur higher organic matter contents and increased soil biological activity [18,19]; however, crop yields are generally lower [16]. Zn concentration in organically grown wheat on low-P soils in Australia was found to be higher, likely associated to increased mycorrhizal colonization, but the yields on organic farms were 17–84% lower [20]. It is well known that yield correlates negatively with grain Zn concentration [21]. Therefore it could be argued that dilution due to higher yield explains the relatively lower Zn concentration seen in conventional Australian farms. Accordingly, a study in Switzerland found no significant differences in grain Zn concentration in organically and conventionally grown wheat at a smaller yield difference of only 14% [22].

In this study we compared organic and conventional wheat cropping systems in terms of grain Zn concentration in a region with low soil Zn availability and where Zn malnutrition is a relevant human health problem. While in a first step soil extractable Zn concentration, wheat yield, and grain Zn concentration are compared between the two systems, in a second step the variability is explored through multiple linear regressions. We thus present possible drivers of extractable Zn concentration, wheat yield, and wheat grain Zn concentration. Our hypotheses were that organic cropping systems had: 1) higher extractable soil Zn concentrations, 2) lower yields, and 3) higher grain Zn concentrations.

## Methods

### Site description

**Fig 1:**
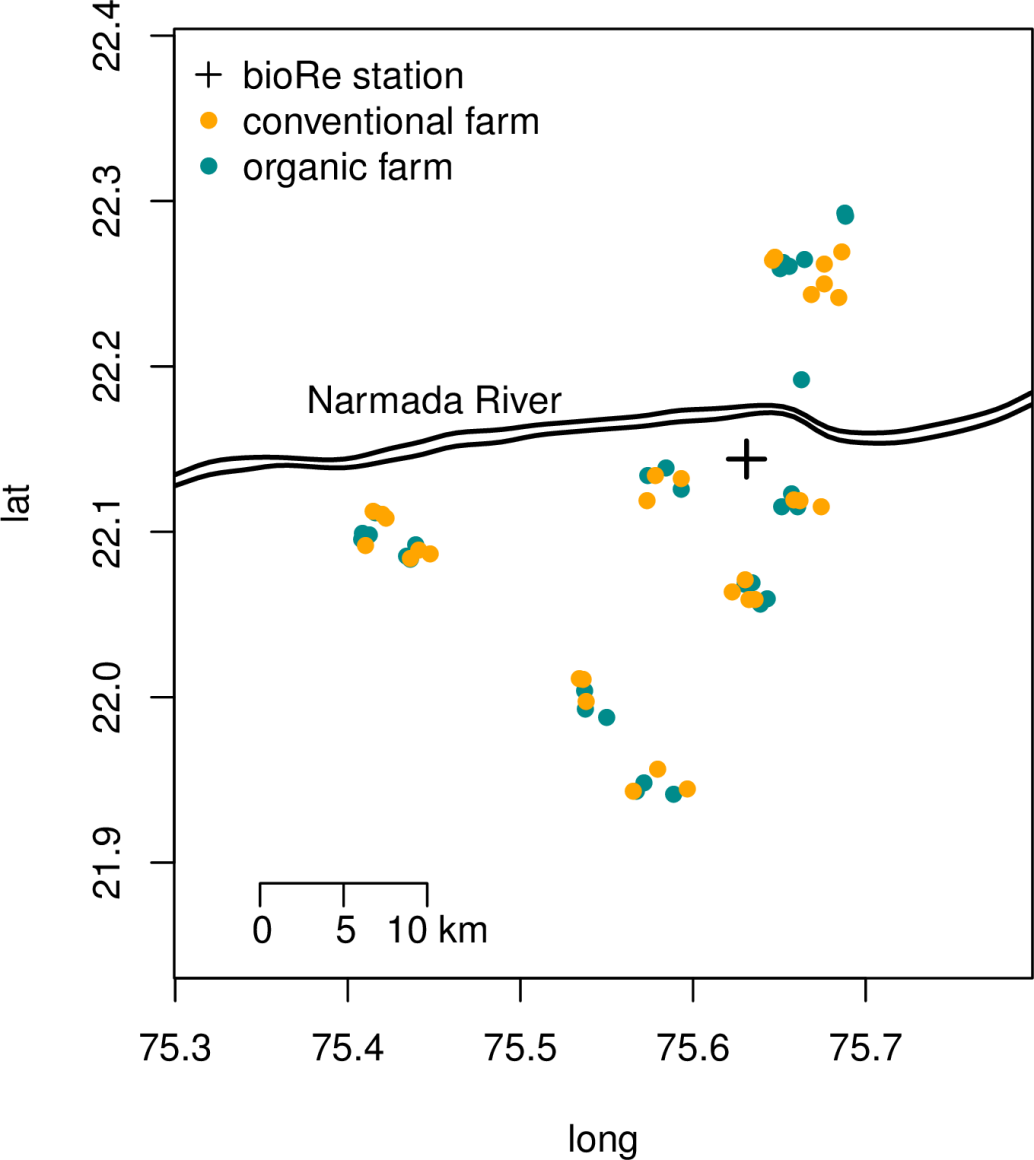
Spatial distribution of farms. Location of 30 organic and 30 conventional farms and bioRe station in study region.

The Nimar District of Madhya Pradesh, India (Fig 1) was chosen for this study due to a well-established network of organic farmers, and the importance of wheat production in the area. According to UNICEF and the World Health Organization, Madhya Pradesh has childhood stunting levels of 50% and the highest prevalence of wasting (children under 5 whose weight is more than 2 standard deviations below the global median) in India [23,24]. Agricultural soils, mostly Vertisols, in Nimar District are known to have a high pH and thus relatively low Zn availability of around 0.57 mg kg^-1^ [25], while the threshold for Zn deficiency in India has been set at <0.6 mg kg^-1^ [5]. Furthermore, bioRe, a cooperative of around 4000 organic farmers, is located in the study area (www.bioreindia.com) and provides information and training on improved organic crop management. Compliance with internationally accepted certification standards and frequent visits by bioRe staff ensure that organic practices are appropriately followed on the registered organic farms. In 2007 a long-term field experiment was established at bioRe’s research station, which compares conventional and organic cropping systems [26]. An earlier study characterized soil characteristics, yield and economic performance of 170 cotton fields in the area [27]. With a series of canals, water reservoirs and wells, irrigation can be practiced even far from the Narmada River, which is the primary source of water in the area. Most farms are between 1 and 10 ha in size; farm work, including soil preparation, weeding, and harvesting, is mainly done by hand or with bullocks [27]. While cotton and soybean are the most important Kharif (monsoon season) crops, in Rabi (winter) most farmers grow wheat.

Sampled farms were selected to be representative of farms in the case study region, and with no systematic differences in organic and conventional farm locations. To minimize soil and management heterogeneity, only fields in the lowlands and with good access to irrigation were included. With respect to these prerequisites, the bioRe research and extension team assembled a list of 50 conventional and 50 organic farmers from six extension centers, in a 25 km radius around bioRe station. From that list, 30 farmers of each practice were selected randomly (Fig 1).

### Soil and wheat sampling and yield determination

Soil samples (top 25 cm) were taken during wheat sowing (November-December 2014). On each field, 10 soil cores were collected on a Z-shaped transect spanning the whole field, and combined into one composite sample, which was air-dried at 40°C, crushed, sieved (2mm) and then transported for lab analysis. Grains were sampled at the time of harvest in March-April 2015. Since not all of the farms could be reached at the same time, some farmers already harvested wheat prior to the second visit. In that case, the farmers set aside 100 g of grain sample. If the crop was still standing but ready to be harvested, 5–7 ears were collected from each of the same 10 locations where soils had been sampled. Each farmer reported the mass of grains harvested on the given field. The field area was measured using the “area calculation” application of a mobile GPS device (Dakota 10, Garmin, Schaffhausen, Switzerland). Dividing the reported mass with the measured field area served as an estimate for the yield.

### Soil analysis

The soil samples were analyzed for pH, soil texture, total element concentrations (C, N, P, K, Ca, S, Mg, Mn, B, Fe, Zn, Cu, Mo, Ni), DTPA extractable micronutrients (Mn, Fe, Zn and Cu), available P and exchangeable K. The pH was measured with 0.01 M CaCl_2_ on < 2 mm soil [28]. Particle size distribution was measured with the laser diffraction method on < 2mm soil. After oxidation with 12.8 M hydrogen peroxide until bubbling stopped, 0.3 g subsamples of soil were submersed in 6 ml of 0.2% Calgone solution and shaken for 2 h [29]. The samples were then analyzed with LS13320 (Beckman Coulter, Bea, USA) using the optical model parameters suggested for soils [30].

Total N was measured on finely ground soil by dry combustion using an NCS analyzer (Flash EA 1112 Series, Thermo Scientific, USA). Inorganic and organic (total minus inorganic) C were measured by a combustion unit (SSM-5000A, Shimadzu, Kyoto, Japan) coupled with a total organic carbon analyzer (TOC-L, Shimadzu, Kyoto, Japan). For determining other total element concentrations, 1 g of finely ground soil was digested with 8 mL aqua regia (HCl and HNO_3_ in a molar ration of 3:1) at 90°C for 120 min and run through ICP-OES (Varian, Palo Alto, USA) [31]. International Soil-Analytical Exchange standard soils 910 and 952 were used as references (http://www.wepal.nl/). Extractable Zn was determined by extracting 10 g of finely ground soil with 20 ml of 0.005 M DTPA and 0.1 M TEA (triethanolamine) followed by measurement on an ICP-OES [32]. The amount of P extractable by the Olsen method was taken as a proxy for available P and measured following the protocol of Schoenau and O’Halloran (2007) [33]. After extraction with 0.5 M NaHCO_3_, the samples were measured in a spectrophotometer (50 Scan, Varian, Palo Alto, USA) at a wavelength of 880 nm against a P-standard series with known P concentrations and also containing 0.5 M NaHCO_3_. Exchangeable K was extracted with neutral ammonium acetate [34] and measured on an ICP-OES.

### Grain analysis

Grains were pulverized in ceramic capsules with a mixing mill (MM200, Retsch, Haan, Germany) by rotating for 1 minute at a frequency of 30 Hz. Total N and total C concentrations were measured by dry combustion using an NCS analyzer (Flash EA 1112 Series, Thermo Scientific, USA). Protein concentration was calculated by multiplying grain N concentration by a conversion factor of 5.7 [35]. Concentrations of other elements were measured by ICP-OES after microwave digestion. One mL of 14.4 M HNO_3_ and 2 mL of 9.8 M H_2_O_2_ were added to subsamples of 100 mg finely ground wheat grain. The samples were then digested for 30 minutes at a pressure of 40 bar and temperature up to 240°C (turboWave, MLS GmbH, Leutkirch, Germany). International Plant-Analytical Exchange references 783 or 197 were included in each digestion set and used to validate the results (http://www.wepal.nl/).

### Interviews

During the time of wheat sowing (November-December 2014), structured face-to-face interviews were conducted with the farmers. The interview included questions on farming practice, training in nutrient management, livestock number, use and amounts of farmyard manure, and wheat cultivar used (Table 1). The exact interview questions can be found in S1 Table. During the time of wheat harvest, some fields were still green, while others were already being harvested. Thus harvest date was noted during field visits and through talking to farmers. In March and April the weather becomes persistently hotter and drier [26]. Our assumption was that late harvest date, i.e. growth period shifted in time, implied increased likelihood of drought exposure.

**Table 1:** List of variables used and how each variable was measured. If a hypothesis existed why a variable might be a predictor for a given response variable, the variable was included in the maximum scope of the respective model.

			max. scope of model:		
indicator	unit	method of assessment	DTPA^a^ Zn	yield	grain Zn
pH	-	measured in 0.01 M CaCl2	x	x	x
clay	g kg^-1^	laser diffraction	x	x	
silt	g kg^-1^	laser diffraction		x	
inorganic C	g kg^-1^	dry combustion with TOC-L analyzer	x		
organic C	g kg^-1^	dry combustion with TOC-L analyzer	x	x	x
N	g kg^-1^	dry combustion with NCS analyzer	x	x	x
P	mg kg^-1^	digestion with aqua regia	x		
S	mg kg^-1^	digestion with aqua regia	x		
Zn	mg kg^-1^	digestion with aqua regia	x		x
DTPA Zn	mg kg^-1^	DTPA-extraction		x	x
available P	mg kg^-1^	Olsen's P extraction with NaHCO_3_	x	x	x
exchangeable K	mg kg^-1^	extraction with NH_4_OAc		x	
cropping system	[ORG/CONV]	farmer interview	x	x	x
cultivar	-	farmer interview		x	x
yield	kg ha^-1^	farmer reporting			x
harvest date	day after January 1st	noted on field visits and through talking with farmers		x	
grain protein	g kg^-1^	dry combustion with NCS analyzer			x
training	[yes/no]	farmer interview		x	x
FYM^b^	livestock ha^-1^	farmer interview	x	x	x

^a^DTPA (diethylene triamine pentaacetic acid)-extractable Zn in the soil is a proxy for plant available Zn.

^b^Farmyard manure availability indicator, see text for details.

The production of farmyard manure as an organic fertilizer was estimated by assuming that farmyard manure production was directly proportional to livestock units.

livestockunits=cows+1.7*bullocks+1.7*buffalo+0.3*calves (1)

‘Livestock units’, was calculated based on masses of tropical livestock in relation to a typical dairy cow (250 kg) (http://www.fao.org/ag/againfo/programmes/en/lead/toolbox /Mixed1/TLU.htm). Since farmers in the region are known to also use farmyard manure as fuel and or purchase additional farmyard manure, the availability of farmyard manure (in livestock units ha^-1^) was estimated by considering these variables.

$$FYM=\frac{livestock\;units*(1-proportion\;farmyard\;manure\;used\;as\;fuel+proportion\;additional\;purchase)}{area\;of\;arable\;land}$$ (2)

Farmers were asked to estimate proportion of farmyard manure used as fuel and additional purchases of farmyard manure, if any, during interviews. While the FYM indicator is not a very precise measurement, in such a complex system with incomplete knowledge it was judged to be the best approximation of farmyard manure input to the fields. The FYM indicator cannot be expected to crisply depict reality, but we consider it to be sensitive to large trends or systematic differences between farming systems compared in this study.

### Statistical analysis and modeling

Student’s *t*-test was applied to determine numeric differences between organic and conventional farms. Non-normal data were log-transformed to meet the assumption of normality. Before transforming to logarithms, the value 1 was added to the FYM indicator. Multiple linear regression models were fitted to the three response variables "extractable Zn in soil", "wheat yield", and "wheat grain Zn concentration" by a stepwise procedure. For each response, we derived from our hypotheses a "maximum-scope" model that included all continuous explanatory variables that we expected to influence the response, along with their interactions with the categorical explanatory variables "training," "cropping system," and “cultivar” (Table 1). The R function step [36] was then used for stepwise selection of explanatory variables by minimizing Akaike's information criterion (AIC) [37]. AIC rewards goodness of fit but penalizes model complexity. Stepwise selection of explanatory variables has been used in a similar study modeling grain Zn, Cu and Fe concentrations with soil and climate variables in Iran [38]. As we observed a non-monotonic increase of yield with exchangeable K, we added a quadratic term to the yield model. Two observations with especially high extractable soil Zn (> 2 mg kg^-1^) were removed for data analysis because we suspected Zn contamination during analysis. All statistical tests were conducted at the significance level *p* < 0.05.

### Ethical approval

The farmer interview study has received ethical approval by the Ethics Commission of the Swiss Federal Institute of Technology in Zürich (study number EK 2016-N-30). All interviewees provided their informed written consent. Furthermore, soil and grain samples were collected in participation with the farmers and upon their prior permission.

## Results

### Interviews

The interviews revealed that farming practices differed only subtly between organic and conventional farms in the case study region. For both organic and conventional farms the wheat cultivar Lok-1 was most often used (CONV: 56%, ORG: 44%), followed by HI-1544 (CONV: 14%, ORG: 20%), GW-366 (CONV: 8%, ORG: 7%), GW-322 (CONV: 6%, ORG: 5%), and WH-147 (CONV: 6%, ORG: 5%). FYM, the farmyard manure availability indicator, was 3.50 and 3.30 livestock units ha^-1^ for organic and conventional farms, respectively (*t* = -0.98, *p* = 0.33) (Table 2). Seven organic and four conventional farmers reported purchasing additional farmyard manure, and purchases ranged from 17–66% of the farm’s own farmyard manure production. Average harvesting dates were also similar for organic and conventional farms (*t* = -0.78, *p* = 0.45). However, only 23% of conventional farmers, compared to 93% of organic farmers stated to have received training for managing nutrients. Training had mostly been conducted by bioRe extension services and included courses on manure management and composting.

**Table 2:** Means, standard errors, t-test statistics, and p-values for soil, wheat grain and management variables of 30 organic (ORG) and 30 conventional (CONV) farms in the study region. Significant differences (p < 0.05) are marked in bold. SEM = standard error of the mean.

	CONV		ORG		t-test	
	mean	SEM	mean	SEM	statistic	p-value
pH	7.20	0.032	7.15	0.050	0.791	0.43
clay [g kg^-1^]	359	22.4	396	20.4	-1.24	0.22
silt [g kg^-1^]	539	14.1	521	14.2	0.949	0.35
inorganic C [g kg^-1^]^a^	2.95	0.791	1.81	0.417	0.501	0.62
organic C [g kg^-1^]	6.44	0.276	6.96	0.315	-1.24	0.22
N [g kg^-1^]	0.634	0.023	0.621	0.027	0.382	0.70
P [g kg^-1^]	0.743	0.054	0.638	0.046	1.48	0.15
S [g kg^-1^]	0.134	0.0090	0.116	0.0058	1.73	0.09
Zn [mg kg^-1^]	124	4.18	121	3.83	0.400	0.69
DTPA Zn [mg kg^-1^]^b^	0.634	0.058	0.642	0.042	-0.515	0.61
available P [mg kg^-1^]^a^	4.07	0.475	3.29	0.516	1.42	0.16
exchangeable K [mg kg^-1^]^a^	266	20.2	258	19.8	0.448	0.66
grain Zn [mg kg^-1^]^a^	**27.6**	**1.16**	**32.1**	**1.66**	**-2.22**	**0.03**
grain protein [g kg^-1^]	121	2.16	129	3.28	-1.94	0.06
grain S [g kg^-1^]	**1.53**	**0.0382**	**1.65**	**0.0446**	**-2.04**	**0.05**
yield [kg ha^-1^]	3370	181	3350	137	0.08	0.94
harvest date [day]	74.7	1.80	76.9	2.13	-0.766	0.45
FYM^a^^,^^c^	3.30	0.660	3.50	0.435	-0.975	0.33

^a^t-test performed on log-transformed data to meet assumption of normality

^b^DTPA (diethylene triamine pentaacetic acid)-extractable Zn, proxy for plant-available Zn in the soil

^c^Farmyard manure availability indicator in livestock units ha^-1^

### Soils

**Fig 2:**
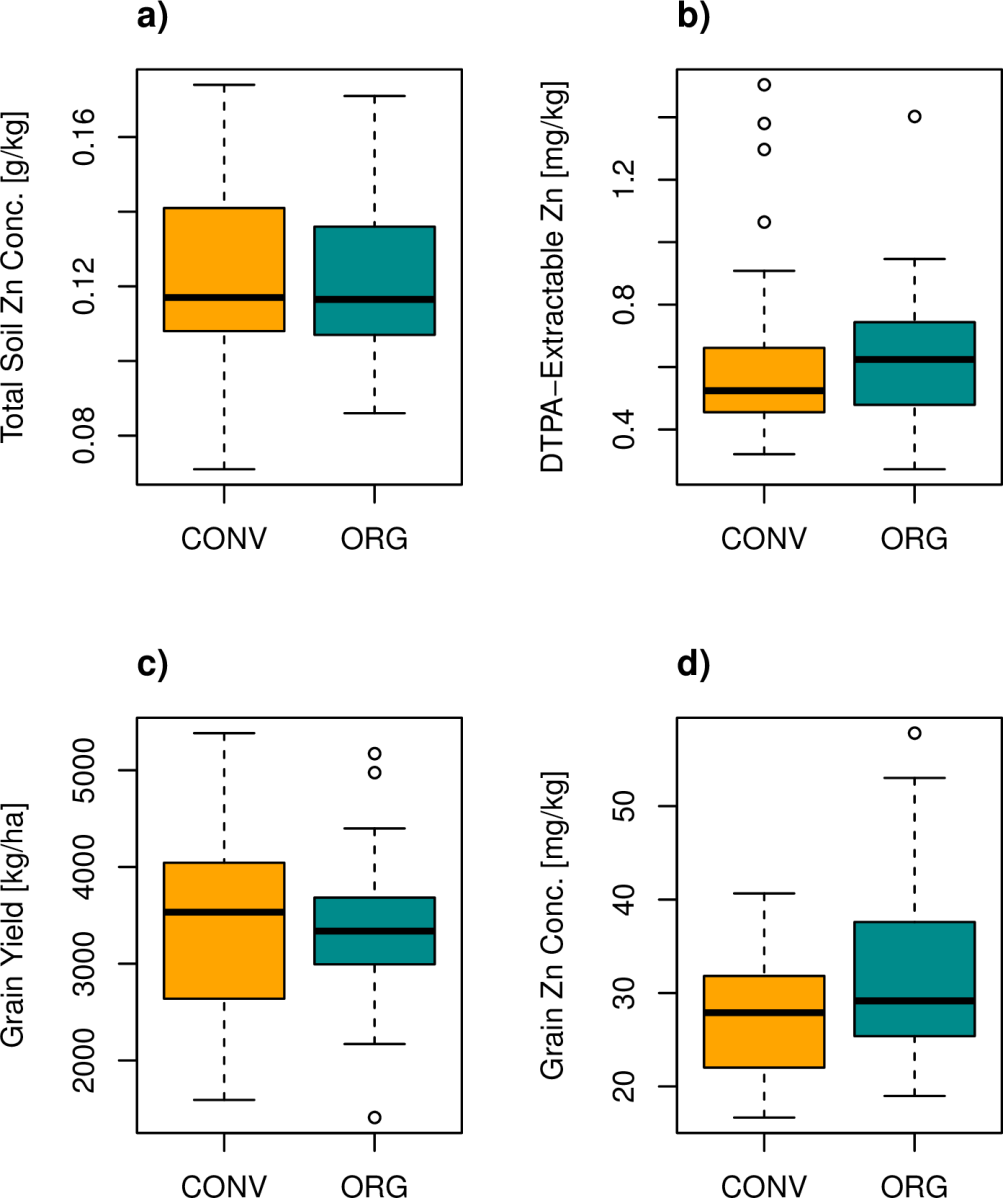
**Boxplots of a) total soil Zn, b) DTPA (diethylene triamine pentaacetic acid)-extractable soil Zn concentration, c) wheat yield, and d) wheat grain Zn concentration for 30 organic and 30 conventional farms.** Thick black lines represent medians.

Total and extractable Zn concentrations in the soil did not differ significantly between organic and conventional systems (Fig 2A and 2B). Mean total soil Zn was around 122 mg kg^-1^ for both systems, with a standard deviation of 21.8 mg kg^-1^. Despite the large pool of total Zn, only 0.64 ± 0.27 mg kg^-1^ was DTPA-extractable. According to the optimal regression models, total soil Zn and total soil S best predicted DTPA-extractable Zn and explained 25% of the variability in the response (Table 3). Inclusion of management variables, cropping system or FYM, did not improve the model.

**Table 3:** Multiple regression models for extractable soil Zn, yield, and wheat grain Zn. Models were determined by a step-wise selection process that maximizes Akaike’s Information Criterion. For each response variable (extractable Zn, yield, and grain Zn), one model was fit considering only soil and plant explanatory variables (models 1, 2, and 5). A second model additionally considered appropriate management variables (models 3, 4, 6, and 7). None of the considered management variables improved the extractable Zn model. See Table 1 for a list of explanatory variables included in the maximum scope for each model selection process.

response variable		multiple regression model	adjusted R^2^^a^	F-statistic	significance level
extractable Zn	(1)	log (extractable Zn) = 6.49 (total Zn) + 0.321 log (total S) - 0.637	0.25	10.3	0.0002
Yield	(2)	yield = 530 log (Available P) + 14400 log (Exchangeable K) - 1360 log (Exchangeable K)^2^–2.02 (silt) - 34100	0.24	5.63	0.0007
training yes^b^:	(3)	yield = 242 log (Available P) + 14600 log (Exchangeable K) - 1350 log (Exchangeable K)^2^–2.52 (silt)– 31.3 (harvest date)– 32200	0.42	7.11	< 0.0001
training no^b^:	(4)	yield = 842 log (Available P) + 14600 log (Exchangeable K) - 1350 log (Exchangeable K)^2^–2.52 (silt)– 31.3 (harvest date)– 33000			
grain Zn	(5)	log (grain Zn) = - 9.96 x 10^−2^ log (Available P) + 4.51 x 10^−3^ (grain protein) - 9.45 x 10^−5^ (yield) + 3.23	0.35	11.6	< 0.0001
ORG^c^:	(6)	log (grain Zn) = - 0.132 log (Available P) + 6.10 x 10^−3^ (grain protein) - 8.48 x 10^−5^ (yield) + 9.05 x 10^−2^ log (FYM) + 2.93	0.46	9.31	< 0.0001
CONV^c^:	(7)	log (grain Zn) = - 0.132 log (Available P) - 4.26 x 10^−3^ (grain protein) - 8.48 x 10^−5^ (yield) + 9.05 x 10^−2^ log (FYM) + 4.15			
		** **			

^a^R^2^ was adjusted for the number of predictors and sample size for comparisons among different models

^b^due to a significant training x Available P interaction, the equations are split up for farmers with and without training

^c^due to a significant cropping system x grain protein interaction, the equations are split up for ORG and CONV

Soils of organic and conventional farms did not differ significantly in any of the other measured variables, though conventional soils tended to have higher macronutrient concentrations. The mean pH of both organic and conventional soils was around 7.2 (Table 2). Soil texture was also not significantly different. Both organic and conventionally managed soils had total N concentrations of around 0.63 ± 0.13 g kg^-1^. Mean total P and total S tended to be higher in conventional soils, but were not significantly different to organic. Conventional soils also tended to contain more Olsen P; mean extractable P was 4.1 mg kg^-1^ for conventional and 3.3 for organic (*t* = 1.4, *p* = 0.16). Mean exchangeable K was around 260 ± 110 mg kg^-1^ for both cropping systems. Means, standard errors, and *t*-tests for additional soil parameters can be found in S2–S4 Tables.

### Yield

Organic and conventional farmers attained mean yields of 3350 and 3370 kg ha^-1^, respectively (*t* = 0.08, *p* = 0.94). Conventional farmers who stated to having received training in nutrient management tended to attain higher yields than conventional farmers without training (*t* = -1.5, *p* = 0.16). Despite similar means for organic and conventional farms, the yield of conventional farms tended to be more variable (Fig 2C), although the variances did not differ significantly (Brown-Forsythe test statistic = 3.2, *p* = 0.079; Levene test statistic = 3.8, *p* = 0.055). If only soil variables were considered, a regression model accounting for available P, exchangeable K, and silt gave the best yield predictions with an adjusted R^2^ of 0.24. Including management variables (training and harvest date) further improved the fit to the data (adjusted R^2^ 0.42, Table 3). The model included a significant interaction effect of training with log(available P): yield depended more strongly on log(available P) for untrained compared with trained farmers (Table 3).

### Grains

Wheat grains from organic farms had significantly greater Zn concentrations than conventionally grown wheat grains (*t* = -2.2, *p* = 0.03) (Fig 2D). With 32.1 mg kg^-1^ as compared to 27.6 mg kg^-1^ Zn, wheat grains from organic farms contained on average almost 20% more Zn than wheat grains from conventional farms (Table 2). Wheat grains from organic farms also contained more S (*t* = -2.0, *p* = 0.046) and tended to contain more N and thus also grain proteins (*t* = -1.9, *p* = 0.058). Other elements–C, P, K, Ca, Mg, Fe, Mn, Cu–were present in similar concentrations in grains from both farming systems (S5 Table). A regression model including yield, available P, and grain protein best predicted grain Zn and had an adjusted R^2^ of 0.35. Including management variables (farmyard manure availability and cropping system) improved the model fit (adjusted R^2^ to 0.46, Table 3). The optimal model contained a significant interaction effect of grain protein with cropping system (positive dependence for organic and negative for conventional farms). The farmyard manure availability indicator (FYM) had a positive effect on predicted grain Zn (Table 3), and was significant in the full model (*t* = 2.4, *p* = 0.019). However, as a simple regression FYM was not significant (*F* = 1.7, *p* = 0.19) and explained only 3% of the variability in grain Zn.

## Discussion

The hypothesis that soils under organic cropping would have higher levels of DTPA-extractable Zn was not supported by the results. This may be due to the fact that there was little difference in the use of organic fertilizers between the two types of farms in our survey. Based on the farmyard manure indicator (FYM), availability of FYM on conventional farms was comparable to organic farms. The hypothesis that organic cropping systems would obtain lower yields was also not supported: both systems attained average yields around 3400 kg ha^-1^. The nearby farming systems comparison field experiment reported yield levels of 2800–3300 kg ha^-1^ for organic treatments and 3200–4200 kg ha^-1^ for conventional treatments between 2007–2010 [26]. Probably due to smaller management differences on farms compared to those on the field trial, we did not find this yield gap on farms in the area. Eyhorn et al. [25] also found higher or equal cotton yields of organic compared to conventional farmers in the area, despite lower organic cotton yields on the system-trial [26].

**Fig 3:**
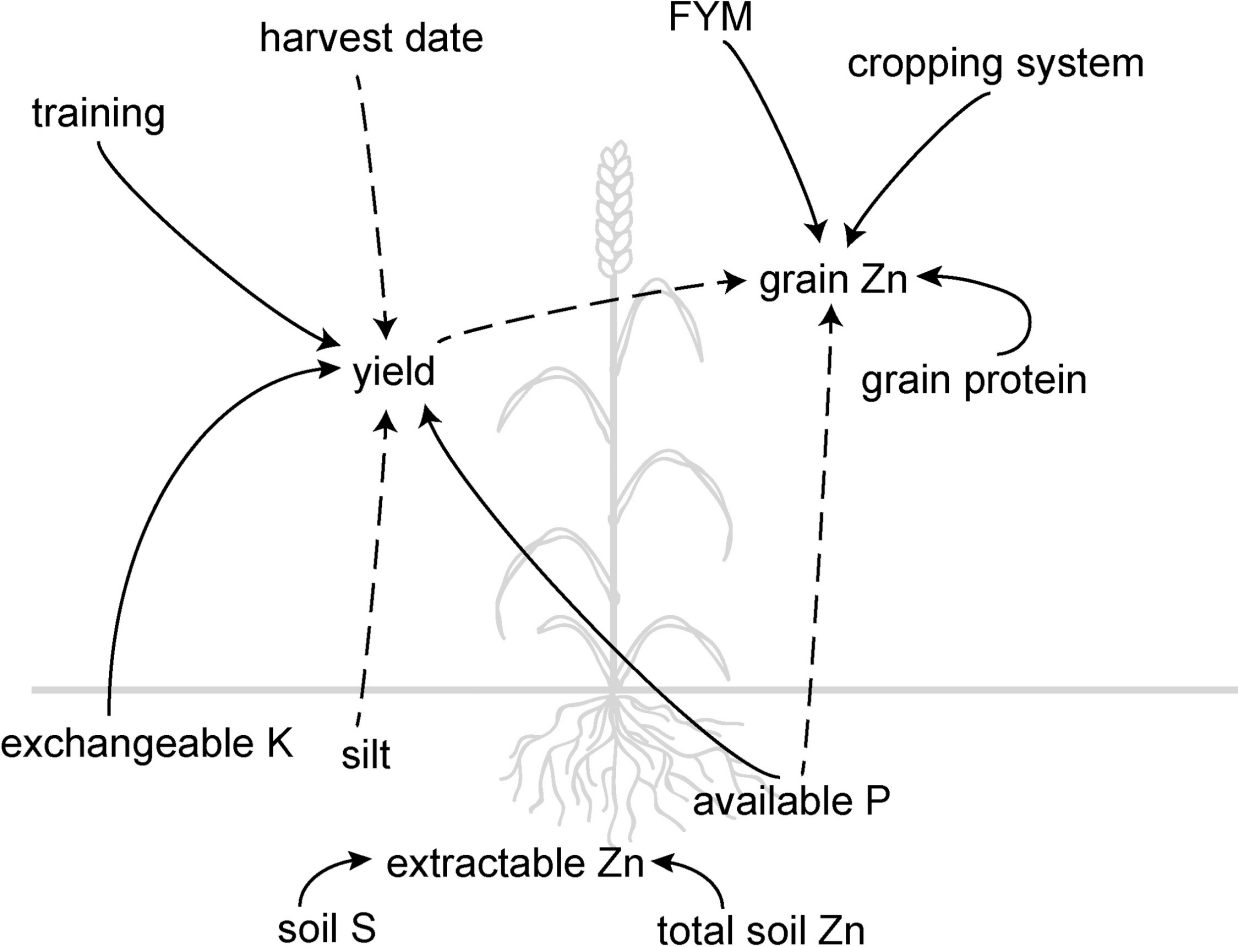
Schematic diagram of correlations as determined by multiple linear regressions in the case study area. Solid lines refer to positive, dotted to negative effects. While soil available P had a positive effect on yield, it was negatively correlated to grain Zn concentration. Yield also has a negative relationship with grain Zn concentration. Organic farmers had improved grain Zn concentrations because they tended to have lower levels of available P in the soil but higher grain protein concentrations. Organic farmers were able to maintain yield levels of conventional farmers by compensating for the lack of chemical fertilizers (lower available soil P levels) with improved nutrient management training.

Despite the similarities in soil conditions, farming practices, and yield level, organic wheat grains contained significantly higher concentrations of Zn. Since Zn is associated with N and S in proteins in grains [39,40], the fact that these elements were also more enriched in organic grains was consistent with the finding of higher grain Zn. The HarvestPlus target is to increase wheat grain Zn in India from a baseline of 25 to 37 mg kg^-1^, while maintaining a “competitive yield” [9]. A multilocational irrigated trial with elite Zn lines in Northern India reported slightly higher average grain Zn concentrations (33.6 ± 0.7 mg kg^-1^) but at lower average yield levels (2700 ± 200 kg ha^-1^) [41] than in our survey here. Organic farmers in our study thus attained statistically significant and nutritionally relevant increases in grain Zn concentration. In the following we discuss how organic farmers could have achieved this increase in grain Zn concentration without compromising yield. The discussion addresses possible underlying mechanisms for the results of our regression analysis, as summarized in Fig 3.

### What influenced extractability of Zn in soils?

**Fig 4:**
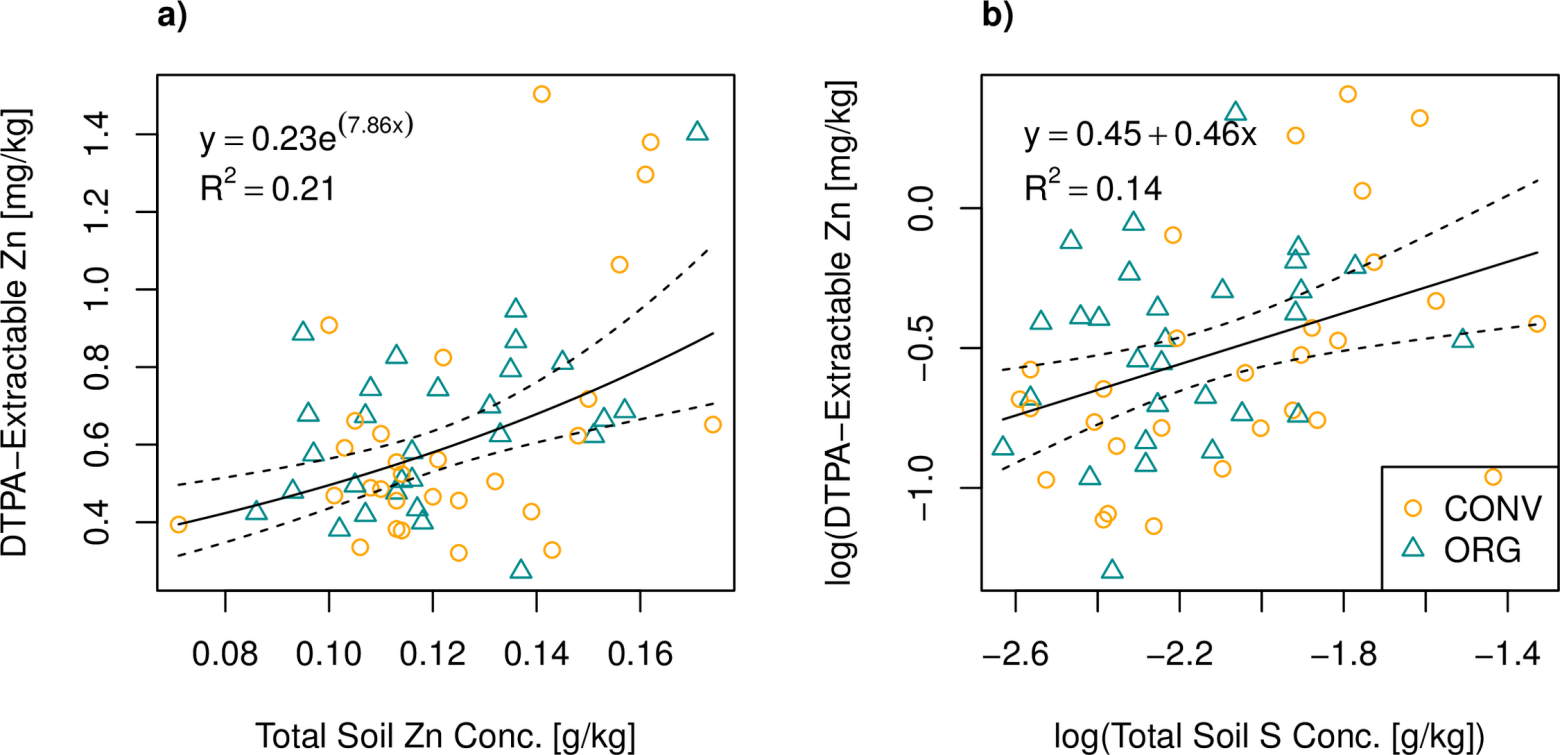
**Simple regressions of log(DTPA-extractable Zn) with a) total soil Zn and b) log(total soil S).** P-values for the regressions were < 0.001 (*F* = 14.8) and 0.003 (*F* = 9.39), respectively. Dotted lines are 95% confidence regions of the regression curves.

Both total soil Zn and total soil S showed positive correlations with extractable soil Zn (Fig 4). The larger the pool of total Zn, the more is also in DTPA-extractable forms. The positive relationship with S is less self-evident. In aerobic soils, the vast majority of S is in organic forms [42], and these S containing organic molecules may also bind Zn [43]. Thus the observed correlation between total soil S and DTPA-extractable Zn suggests that S containing organic molecules are important sources of available Zn in these soils. Neither pH (*F* = 0.50, *p* = 0.48) nor soil N (*F* = 1.5, *p* = 0.22) had a significant correlation with extractable Zn, despite a known pH-dependence of Zn availability [13]. The reason why we saw no pH-effect is probably that all soils were in neutral-slightly alkaline pH range and the dataset had a narrow range of pH values. The first and third quartiles of the pH distribution were 7.01 and 7.32 respectively. Accordingly, pH was also not a significant predictor of wheat yield or grain Zn concentration (Table 3). Also, neither soil organic C nor the FYM indicator were a significant predictor of extractable Zn.

### What influenced wheat yield?

**Fig 5:**
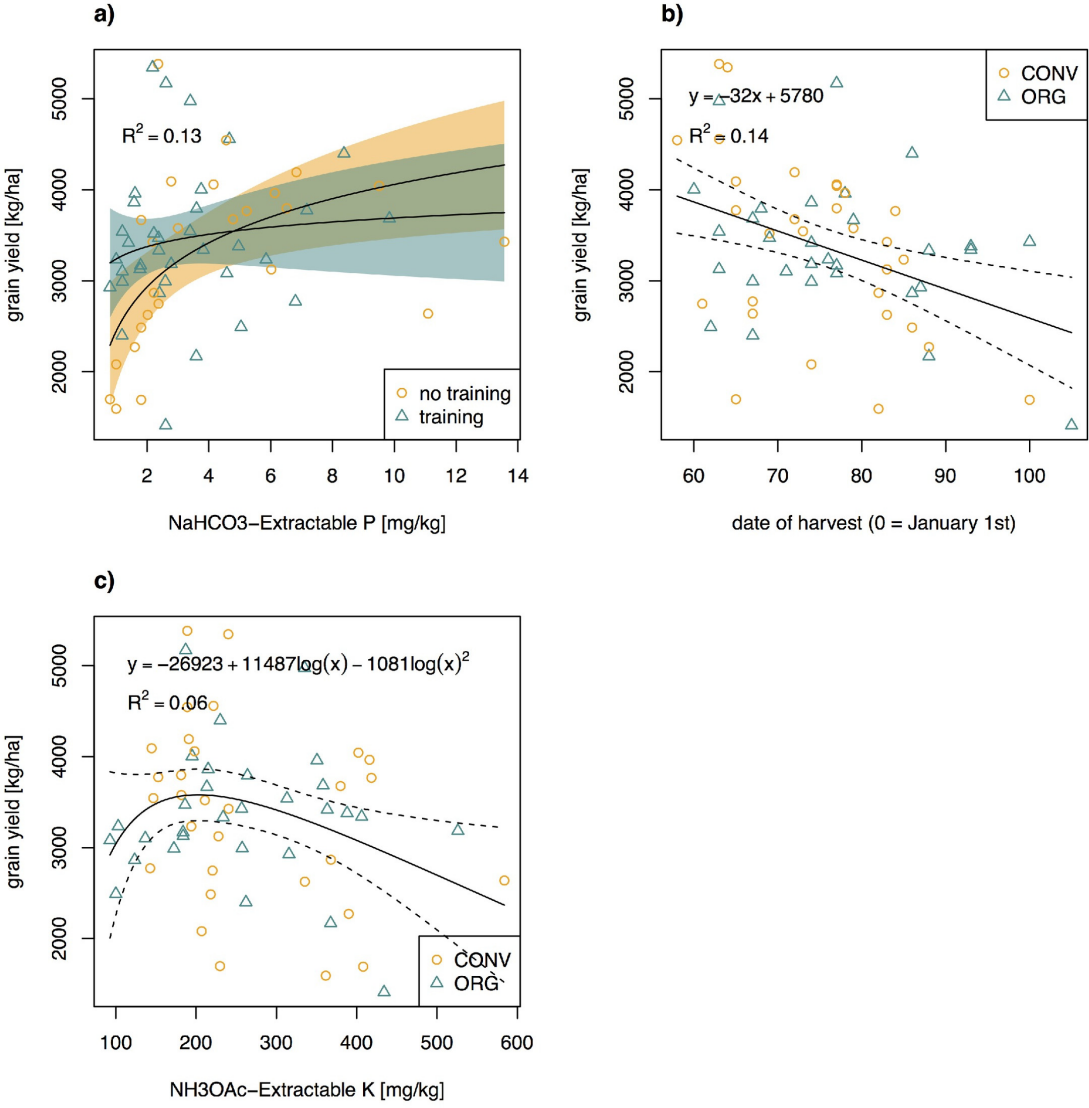
**Regressions of wheat grain yield with a) available P, b) harvest date, and c) exchangeable K.** Available P had a significant interaction effect with training on grain yield. P-values for the regressions were 0.01 (*F* = 3.99), 0.002 (*F* = 10.7), and 0.06 (*F* = 2.94), respectively. Dotted lines (shaded area) are 95% confidence regions of the regression curves.

Stepwise selection of explanatory variables revealed that available P, training in nutrient management, harvest date, and exchangeable soil K were the most significant predictors of yield. Our results suggest that P was the main yield-limiting nutrient. In agreement with agronomic guidelines for fertilization (also based on Olsen P measurement), the slope of the yield trend line decreased as available P approached 15 mg kg^-1^ [44]. However, yields of farmers who received training in nutrient management were less sensitive to low P levels (Fig 5A). While our interview questionnaire did not address dissemination of other knowledge, it is likely that “training in nutrient management” also comes with other knowledge exchange, and general improved agronomic practices. Most organic farmers are/have been associated with bioRe, where they receive training and knowledge from time to time, along with appropriate seed and inputs for organic farming. Some of the organic farmers are also involved in the participatory research activities carried out by the innovation platform developed at bioRe in collaboration with the Research Institute of Organic Agriculture, Switzerland [45]. This suggests that organic farmers achieve similar yields to conventional farmers, despite not using chemical fertilizers, because good agricultural practice makes more out of available natural capital. The training effect also results in more homogeneous practices among organic farmers, as can be seen in the lower between-farm variability of yield for organic compared to conventional farmers (Fig 2C).

The negative relationship between yield and harvest date (Fig 5B) is in line with our hypothesis that this variable was a proxy for drought stress. The further the wheat-growing season extended into the hot and dry Zaid season, the more likely the plants suffered from drought. The relationship between yield and exchangeable K (Fig 5C) is less easy to interpret. A positive effect on predicted yield for NH_4_OAc-extractable K values below 150 mg kg^-1^ was expected, since 150 mg kg^-1^ is considered as the threshold below which an application of K fertilizer will increase yields [46]. Though values between 250–800 mg kg^-1^ are considered high, we have no reason to suspect phytotoxic effects at these levels. The low-yield points pulling the regression down for low K values are most likely due to other farm characteristics rather than a direct exchangeable K effect.

### What influenced grain Zn concentration?

**Fig 6:**
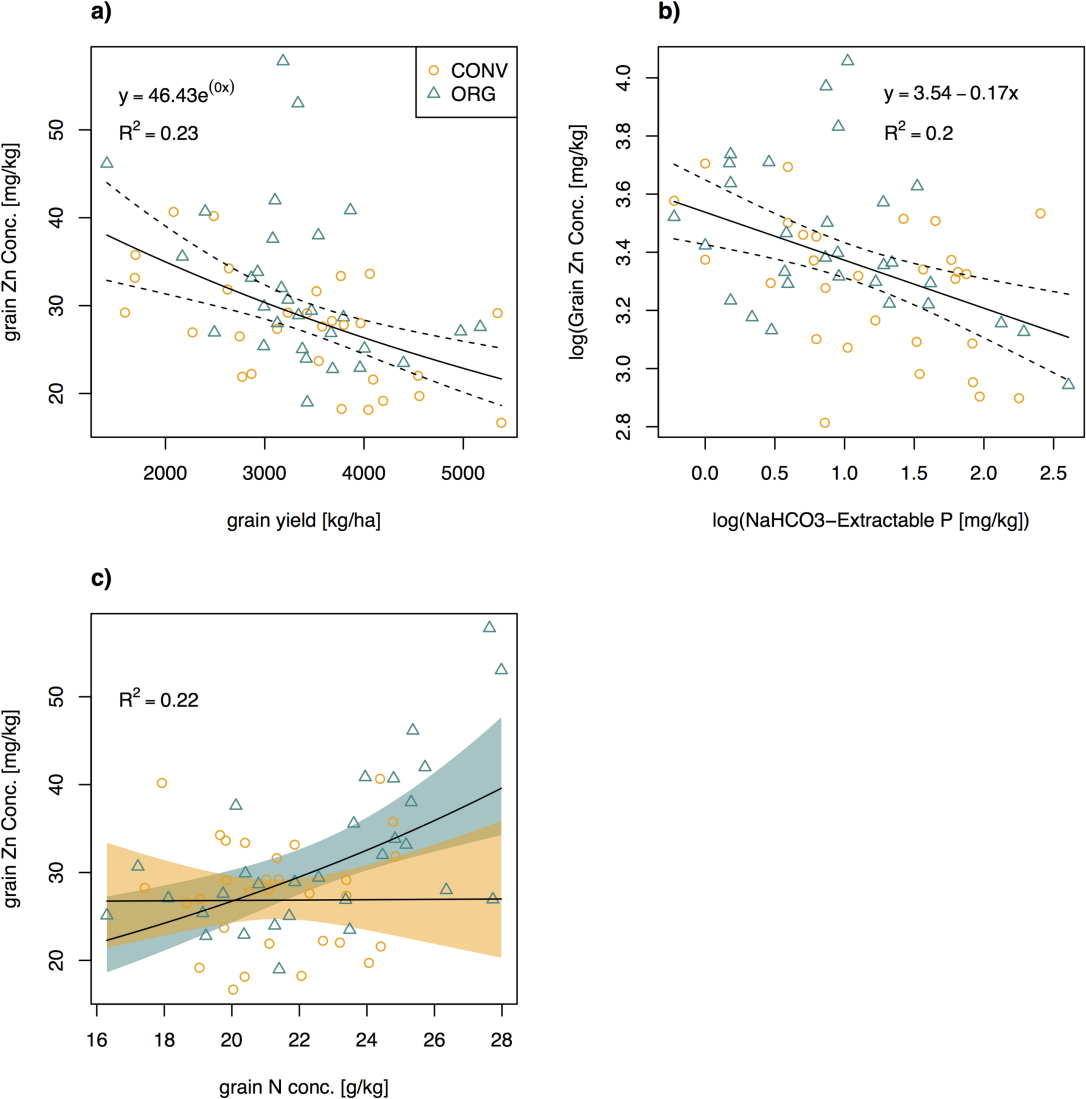
**Regressions of wheat grain Zn concentration with a) grain yield, b) available P, and c) grain protein concentration.** Grain protein concentration had a significant interaction with cropping system on grain Zn concentration; orange shading refers to conventional. P-values for the regressions were all < 0.001 and *F*-statistics were 17.3, 14.4, and 6.60, respectively. Dotted lines (shaded area) are 95% confidence regions of the regression curves.

Decreasing grain Zn concentrations associated with increasing grain yields are often interpreted as the result of a dilution effect due to a larger proportion of tissue with low Zn density [21]. Vice versa, an increase in grain Zn concentration may just be a result of a decrease in yield. For example, a study in southeastern Australia showed that organic wheat cropping may increase grain Zn by 25–56%, but at the expense of a 17–84% decrease in yield [20]. Also in our study yield showed a strong negative correlation with grain Zn concentration, but there was no yield difference between organic and conventional wheat (Fig 6A). Thus, the dilution effect does not explain the difference in grain Zn between the two cropping systems. Aside from yield, available soil P also correlated negatively with grain Zn (Fig 6B), even if the yield effect was considered (Table 3). A negative correlation of grain Zn with available P was also observed in wheat across three provinces in Iran [38]. In the latter study, Olsen P values were much higher than in our study, ranging from 10 to 165 mg kg^-1^. One reason for P-induced Zn deficiency can be that mycorrhizal colonization is reduced with increasing soil P levels [47]. Mycorrhizae can make an important contribution to plant Zn nutrition on soils with low Zn solubility [48]. Higher abundance of mycorrhizal root colonization in organic than conventional farming explained higher grain Zn concentration in field trials in southeastern Australia [20]. The conventional farms in the latter study applied 16–18 kg P ha^-1^, which is similar to the rates of P application of conventional farmers in our study (data not shown). However, since soils in our study were low in P (Olsen P values at 1–14 mg kg^-1^), and we do not know of any studies showing P-inhibition of arbuscular mycorrhizal fungi at this level, we cannot explain the underlying mechanism behind the observed grain Zn–soil available P relationship.

Considering that grain protein was the only variable to have an interaction effect with cropping system, N nutrition seems to be key to understanding differences in grain Zn concentration between the two systems. Proteins act as a sink for Zn in grains [40]. While the model with only soil and plant predictors showed a positive correlation between grain Zn and protein, by including management variables the positive correlation was only observed for organic farms (Table 3)(Fig 6C). While conventional systems are usually less N-limited, we did not detect any differences in total soil N (Table 2). Despite similar amounts of soil N, N uptake by wheat grains tended to be greater on organically managed fields (Table 2). Similar to what was seen for P, this signifies a higher nutrient efficiency for organic wheat. Nutrient efficiency, not to be confused with nutrient use efficiency, is defined by differences in relative growth or yield under nutrient-limiting conditions [49]. While usually nutrient efficiency is used to compare different cultivars, we did not find any significant cultivar effects. Instead, sociological (training) and management (cropping system) variables affected yield response curve to P and grain Zn accumulation, respectively (Table 3). Higher nutrient efficiency was also seen for S, where organic farms had significantly more grain S despite tending to have lower soil S concentration (Table 2). A possible underlying mechanism could be soil biology, as it is generally accepted that organically managed soils support more active soil biology [18,19], and that N cycling in soils is largely driven by biotic factors [50]. Since N does not seem to be limiting yield (no correlation between soil N and yield), additional N lead to a relative increase in protein concentration. Higher Zn concentration is thus a byproduct of higher protein content in grains.

### Implications

In this study we integrated soil and plant measurements with management and sociological variables to model DTPA-extractable Zn, yield, and grain Zn concentration. As can be seen in (Table 3), inclusion of management variables improved the yield and grain Zn prediction models. None of the soil or plant variables were able to explain why organic farmers would achieve the same yield level as conventional despite lower available P levels. The training factor suggested that there was a human capital effect on organic farmer’s yield. The harvest date variable also improved prediction of yield, as it served as a proxy for not easily-measurable field data. Unfortunately, we did not have the possibility to quantify soil moisture variables, e.g. by means of tensiometers, despite knowing that water stress would significantly influence yield in these irrigated agricultural systems. Harvest date was a simple and quantifiable indicator that likely captured some of this influence. In a study on biophysical factors determining copper, iron and Zn in wheat grains in central Iran, linear regression on soil and climate variables explained 26% of the variability in grain Zn concentration [38]. Karami et al. [38] attributed some of the uncaptured variability to differences in management. In this study we were able to determine management variables in farmer interviews and showed that the addition of management variables to measured field variables did indeed improve linear modeling (Table 3). However, interview answers were often very subjective or riddled with uncertainty. It proved valuable to ask several related questions and see if the answers were correlated, which would suggest accurate answers (results not shown). Also, it was decided to focus on less subjective interview results, such as number of livestock per farm, which every farmer could answer accurately.

The results underline the importance of farmer training and organization. While we found no yield difference between organically and conventionally grown wheat, the local system comparison trial reported that organic were significantly lower than conventional wheat yields [26]. This discrepancy between field and on-farm trials also came forth in a meta-analysis, which reported that in on-farm studies organic yields were on average 88% of conventional yields, compared to an average of only 81% in field trials [16]. While field trials control for possible human capital effects (i.e. the same persons with the same know-how manage all treatments), in on-farm reality organic and conventional farmer populations may have different motivations, beliefs, and know-how [27]. Especially in developing countries, training received upon certification is often highly valued by farmers [51]. In our study, nutrient management training significantly increased predicted yield (Table 3). Better training of conventional farmers would thus likely increase conventional farmer yields to levels reported in the system comparison trial (up to 4200 kg ha^-1^) [26]. However, this would likely come along with reduced grain Zn concentrations due to yield dilution (Fig 6A) if farmers do not adopt more Zn-efficient cultivars and or start applying Zn fertilizers. Field studies in Punjab have shown that Zn fertilization may increase both crop yield and grain Zn concentration [2,52]. Therefore the spread of Zn-efficient crops coupled with Zn fertilization should be pursued to further increase grain quality. In an assessment of economic performance over two full crop rotation in the system comparison trial, Forster et al. [26] report that overall organic cropping was financially competitive even at lower yield levels. From a human nutritional and a financial point of view, organic wheat cropping is thus a sustainable option for farmers in the region.

## Conclusion

To our knowledge, this is the first study reporting increased grain Zn concentrations in organic compared to conventional wheat cropping, where the biofortification effect is not due to decreased yield. Yields for both systems were on average around 3400 kg ha^-1^ and both organic and conventional soils contained similar amounts of total and extractable Zn. Considering that farming practices and soil Zn conditions were similar, the benefit of organic farming could not be explained simply by soil Zn concentration differences. Management variables improved model fits and allowed our regressions to explain a higher percentage of the observed variability. With a model considering yield, soil available P, grain N, cropping system and farmyard manure availability, we were able to explain 46% of the variability in grain Zn concentration of the 60 farms. The analyses revealed that training gave organic farmers an edge and allowed them to produce higher quality wheat grains at the same yield level. Organic farmers’ improved agricultural management was seen in a higher nutrient efficiency, as organic grains accumulated more Zn, N, and S despite similar soil nutrient concentrations, and the same yield despite tending to have lower soil available P. Due to higher grain Zn concentration and same yield, organic wheat farming thus has a potential to improve livelihoods in the region.

## Supporting Information

S1 Table Exact interview question and answer type for each interview variable mentioned. Interviews were conducted face-to-face based on a structured, written questionnaire. FYM here stands for farmyard manure.
(DOCX)

S2 Table Soil total macronutrient concentrations [g kg^-1^] of 30 organic (ORG) and 30 conventional (CONV) farms in the study region. Total C and N were determined by combustion using an NCS analyzer (Flash EA 1112 Series, Thermo Scientific, USA). Other element concentrations were measured by digestion with aqua regia and followed by measurement with ICP-OES. Significant differences (*p* < 0.05) between average values in conventional and organic farm soils are marked in bold. SEM = standard error of the mean.
(DOCX)

S3 Table Soil total micronutrient concentrations [mg kg^-1^] of 30 organic (ORG) and 30 conventional (CONV) farms in the study region. Samples were digested with aqua regia and concentrations measured with ICP-OES. SEM = standard error of the mean.
(DOCX)

S4 Table pH, clay content, and available nutrient concentrations of 30 organic (ORG) and 30 conventional (CONV) farms in the study region. pH was measured in 0.1 M CaCl_2_ and values are thus lower than if measured in water. Clay content was measured by laser diffraction. Available P was measured using Olsen’s method. Exchangeable K was measured by NH_4_OAc extraction. Available Mn, Fe, Cu and Zn were determined by DTPA-extraction. See Methods section for more details. SEM = standard error of the mean.
(DOCX)

S5 Table Wheat grain nutrient concentrations [mg kg^-1^] of 30 organic (ORG) and 30 conventional (CONV) farms in the study region. Wheat grains were taken during the time of wheat harvest. Total N and total C concentrations were measured by dry combustion using an NCS analyzer (Flash EA 1112 Series, Thermo Scientific, USA). Concentrations of other elements were measured by ICP-OES after microwave digestion. Zn uptake was calculated by multiplying grain Zn concentration by the grain yield. Significant differences (*p* < 0.05) between average values in conventional and organic wheat grains are marked in bold. SEM = standard error of the mean.
(DOCX)

S6 Table CSV file containing all data underlying the findings in this manuscript. (CSV)
