# Supplementary material for: Organic Wheat Farming Improves Grain Zinc Concentration
Source: PLoS One. 2016 Aug 18;11(8):e0160729. doi: 10.1371/journal.pone.0160729 (PMC4990241; doi:10.1371/journal.pone.0160729)
Supplement: S1 Table — Interviews were conducted face-to-face based on a structured, written questionnaire. FYM here stands for farmyard manure. (DOCX) [file pone.0160729.s001.docx]

**S1 Table. Exact interview question and answer type for each interview variable mentioned.** Interviews were conducted face-to-face based on a structured, written questionnaire. FYM here stands for farmyard manure.

| Variable | Exact interview question | Answer type |
| --- | --- | --- |
| cropping system | Which farming practice do you use? | organic/conventional |
| cultivar | What wheat cultivar do you use at the moment? | name of wheat cultivar |
| training | Did you receive training on nutrient management from the cooperative?^a^ | YES/NO |
| livestock | What type(s) and how many animals do you have? | number of cows, bullocks, buffalos, calfs, and goats |
| fuel | What percentage do of the FYM do you use as fuel? | percentage |
| purchase of FYM | Are you using your own animals to produce all the FYM you need? | YES/NO, if no, % that is not self-produced |

^a^pre-question: Are you part of a farmers’ cooperative?
