# Supplementary material for: Organic Wheat Farming Improves Grain Zinc Concentration
Source: PLoS One. 2016 Aug 18;11(8):e0160729. doi: 10.1371/journal.pone.0160729 (PMC4990241; doi:10.1371/journal.pone.0160729)
Supplement: S2 Table — Total C and N were determined by combustion using an NCS analyzer (Flash EA 1112 Series, Thermo Scientific, USA). Other element concentrations were measured by digestion with aqua regia and followed by measurement with ICP-OES. Significant differences (p < 0.05) between average values in conventional and organic farm soils are marked in bold. SEM = standard error of the mean. (DOCX) [file pone.0160729.s002.docx]

**S2 Table. Soil total macronutrient concentrations [g kg^-1^] of 30 organic (ORG) and 30 conventional (CONV) farms in the study region.** Total C and N were determined by combustion using an NCS analyzer (Flash EA 1112 Series, Thermo Scientific, USA). Other element concentrations were measured by digestion with aqua regia and followed by measurement with ICP-OES. Significant differences (*p* < 0.05) between average values in conventional and organic farm soils are marked in bold. SEM = standard error of the mean.

|  |  | CONV | |  | ORG | |  | t-test | |
| --- | --- | --- | --- | --- | --- | --- | --- | --- | --- |
|  |  | mean | SEM |  | mean | SEM |  | statistic | p-value |
| C |  | 11.1 | 0.947 |  | 10.3 | 0.580 |  | 0.731 | 0.47 |
| N |  | 0.634 | 0.023 |  | 0.621 | 0.027 |  | 0.382 | 0.70 |
| P |  | 0.743 | 0.054 |  | 0.638 | 0.046 |  | 1.48 | 0.15 |
| K |  | 1.93 | 0.146 |  | 1.76 | 0.145 |  | 0.812 | 0.42 |
| Ca |  | **18.0** | **1.73** |  | **12.9** | **0.805** |  | **2.67** | **0.01** |
| S |  | 0.134 | 0.0090 |  | 0.116 | 0.0058 |  | 1.73 | 0.09 |
| Mg |  | **7.47** | **0.221** |  | **6.78** | **0.208** |  | **2.27** | **0.03** |
