# Supplementary material for: Organic Wheat Farming Improves Grain Zinc Concentration
Source: PLoS One. 2016 Aug 18;11(8):e0160729. doi: 10.1371/journal.pone.0160729 (PMC4990241; doi:10.1371/journal.pone.0160729)
Supplement: S3 Table — Samples were digested with aqua regia and concentrations measured with ICP-OES. SEM = standard error of the mean. (DOCX) [file pone.0160729.s003.docx]

**S3 Table. Soil total micronutrient concentrations [mg kg^-1^] of 30 organic (ORG) and 30 conventional (CONV) farms in the study region.** Samples were digested with aqua regia and concentrations measured with ICP-OES. SEM = standard error of the mean.

|  |  | CONV | |  | ORG | |  | t-test | |
| --- | --- | --- | --- | --- | --- | --- | --- | --- | --- |
|  |  | mean | SEM |  | mean | SEM |  | statistic | p-value |
| B |  | 411 | 12.0 |  | 418 | 10.3 |  | -0.413 | 0.68 |
| Mn |  | 762 | 27.0 |  | 784 | 20.5 |  | -0.657 | 0.51 |
| Fe |  | 43000 | 880 |  | 42600 | 809 |  | 0.371 | 0.71 |
| Zn |  | 124 | 4.18 |  | 121 | 3.83 |  | 0.400 | 0.69 |
| Cu |  | 201 | 8.43 |  | 195 | 7.21 |  | 0.541 | 0.59 |
| Mo |  | 0.229 | 0.014 |  | 0.256 | 0.019 |  | -1.17 | 0.25 |
| Ni |  | 30.7 | 1.37 |  | 33.6 | 1.63 |  | -1.38 | 0.17 |
