# Supplementary material for: Organic Wheat Farming Improves Grain Zinc Concentration
Source: PLoS One. 2016 Aug 18;11(8):e0160729. doi: 10.1371/journal.pone.0160729 (PMC4990241; doi:10.1371/journal.pone.0160729)
Supplement: S4 Table — pH was measured in 0.1 M CaCl2 and values are thus lower than if measured in water. Clay content was measured by laser diffraction. Available P was measured using Olsen’s method. Exchangeable K was measured by NH4OAc extraction. Available Mn, Fe, Cu and Zn were determined by DTPA-extraction. See Methods section for more details. SEM = standard error of the mean. (DOCX) [file pone.0160729.s004.docx]

**S4 Table. pH, clay content, and available nutrient concentrations of 30 organic (ORG) and 30 conventional (CONV) farms in the study region.** pH was measured in 0.1 M CaCl_2_ and values are thus lower than if measured in water. Clay content was measured by laser diffraction. Available P was measured using Olsen’s method. Exchangeable K was measured by NH_4_OAc extraction. Available Mn, Fe, Cu and Zn were determined by DTPA-extraction. See Methods section for more details. SEM = standard error of the mean.

|  |  | CONV | |  | ORG | |  | t-test | |
| --- | --- | --- | --- | --- | --- | --- | --- | --- | --- |
|  |  | mean | SEM |  | mean | SEM |  | statistic | p-value |
| pH |  | 7.20 | 0.032 |  | 7.15 | 0.050 |  | 0.791 | 0.43 |
| clay^a^ |  | 359 | 22.4 |  | 396 | 20.4 |  | -1.24 | 0.22 |
| available P^b,c^ |  | 4.07 | 0.475 |  | 3.29 | 0.516 |  | 1.42 | 0.16 |
| exchangeable K^b,c^ | | 266 | 20.2 |  | 258 | 19.8 |  | 0.448 | 0.66 |
| available Mn^b^ |  | 8.21 | 0.515 |  | 8.66 | 0.497 |  | -0.626 | 0.53 |
| available Fe^b^ |  | 42.1 | 2.17 |  | 42.2 | 2.40 |  | -0.057 | 0.96 |
| available Zn^b,c^ |  | 0.634 | 0.058 |  | 0.642 | 0.042 |  | -0.515 | 0.61 |
| available Cu^b^ |  | 2.95 | 0.168 |  | 3.01 | 0.174 |  | -0.240 | 0.81 |

^a^in g kg^-1^

^b^in mg kg^-1^

^c^t-test performed on log-transformed data to meet assumption of normality
