# Supplementary material for: Organic Wheat Farming Improves Grain Zinc Concentration
Source: PLoS One. 2016 Aug 18;11(8):e0160729. doi: 10.1371/journal.pone.0160729 (PMC4990241; doi:10.1371/journal.pone.0160729)
Supplement: S5 Table — Wheat grains were taken during the time of wheat harvest. Total N and total C concentrations were measured by dry combustion using an NCS analyzer (Flash EA 1112 Series, Thermo Scientific, USA). Concentrations of other elements were measured by ICP-OES after microwave digestion. Zn uptake was calculated by multiplying grain Zn concentration by the grain yield. Significant differences (p < 0.05) between average values in conventional and organic wheat grains are marked in bold. SEM = standard error of the mean. (DOCX) [file pone.0160729.s005.docx]

**S5 Table. Wheat grain nutrient concentrations [mg kg^-1^] of 30 organic (ORG) and 30 conventional (CONV) farms in the study region.** Wheat grains were taken during the time of wheat harvest. Total N and total C concentrations were measured by dry combustion using an NCS analyzer (Flash EA 1112 Series, Thermo Scientific, USA). Concentrations of other elements were measured by ICP-OES after microwave digestion. Zn uptake was calculated by multiplying grain Zn concentration by the grain yield. Significant differences (*p* < 0.05) between average values in conventional and organic wheat grains are marked in bold. SEM = standard error of the mean.

|  |  | CONV | |  | ORG | |  | t-test | |
| --- | --- | --- | --- | --- | --- | --- | --- | --- | --- |
|  |  | mean | SEM |  | mean | SEM |  | statistic | p-value |
| C^a^ |  | 418 | 0.541 |  | 418 | 0.723 |  | -0.495 | 0.62 |
| N^a^ |  | 21.3 | 0.378 |  | 22.7 | 0.575 |  | -1.94 | 0.06 |
| P |  | 3190 | 89.6 |  | 3120 | 90.9 |  | 0.596 | 0.55 |
| K |  | 3890 | 69.5 |  | 3840 | 93.6 |  | 0.385 | 0.70 |
| Ca |  | 425 | 29.5 |  | 386 | 11.7 |  | 1.20 | 0.23 |
| S |  | **1530** | **38.2** |  | **1650** | **44.6** |  | **-2.04** | **0.05** |
| Mg |  | 1330 | 23.9 |  | 1320 | 27.8 |  | 0.276 | 0.78 |
| Fe |  | 32.1 | 1.40 |  | 32.9 | 1.56 |  | -0.365 | 0.72 |
| Mn |  | 36.6 | 1.19 |  | 35.9 | 1.07 |  | 0.466 | 0.64 |
| Zn^b^ |  | **27.6** | **1.16** |  | **32.1** | **1.66** |  | **-2.23** | **0.03** |
| Cu |  | 4.99 | 0.118 |  | 5.15 | 0.195 |  | -0.690 | 0.49 |
| Zn uptake^b,c^ |  | **89.4** | **4.42** |  | **104.9** | **5.56** |  | **-2.18** | **0.03** |

^a^in g kg^-1^

^b^t-test performed on log-transformed data to meet assumption of normality

^c^in g ha^-1^
